# Machine learning models for diagnosing Alzheimer’s disease using brain cortical complexity

**DOI:** 10.3389/fnagi.2024.1434589

**Published:** 2024-10-09

**Authors:** Shaofan Jiang, Siyu Yang, Kaiji Deng, Rifeng Jiang, Yunjing Xue

**Affiliations:** ^**1**^Department of Radiology, Fujian Medical University Union Hospital, Fuzhou, China; ^2^Fujian Key Laboratory of Intelligent Imaging and Precision Radiotherapy for Tumors, Fujian Medical University, Fuzhou, China; ^3^Department of Neurology, Fujian Medical University Union Hospital, Fuzhou, China; ^4^Fujian Institute of Geriatrics, Fujian Medical University Union Hospital, Fuzhou, China; ^5^Institute of Clinical Neurology, Fujian Medical University, Fuzhou, China

**Keywords:** Alzheimer’s disease, Montreal Cognitive Assessment, machine learning, apolipoprotein E, magnetic resonance imaging

## Abstract

**Objective:**

This study aimed to develop and validate machine learning models (MLMs) to diagnose Alzheimer’s disease (AD) using cortical complexity indicated by fractal dimension (FD).

**Methods:**

A total of 296 participants with normal cognitive (NC) function and 182 with AD from the AD Neuroimaging Initiative database were randomly divided into training and internal validation cohorts. Then, FDs, demographic characteristics, baseline global cognitive function scales [Montreal Cognitive Assessment (MoCA), Functional Activities Questionnaire (FAQ), Global Deterioration Scale (GDS), Neuropsychiatric Inventory (NPI)], phospho-tau (p-tau 181), amyloidβ-42/40, apolipoprotein E (APOE) and polygenic hazard score (PHS) were collected to establish multiple MLMs. Receiver operating characteristic curves were used to evaluate model performance. Participants from our institution (*n* = 66; 33 with NC and 33 with AD) served as external validation cohorts to validate the MLMs. Decision curve analysis was used to estimate the models’ clinical values.

**Results:**

The FDs from 30 out of 69 regions showed significant alteration. All MLMs were conducted based on the 30 significantly different FDs. The FD model had good accuracy in predicting AD in three cohorts [area under the receiver operating characteristic (ROC) curve (AUC) = 0.842, 0.808, and 0.803]. There were no statistically significant differences in AUC values between the FD model and the other combined models in the training and internal validation cohorts except MoCA + FD and FAQ + FD models. Among MLMs, the MoCA + FD model showed the best predictive efficiency in three cohorts (AUC = 0.951, 0.931, and 0.955) and had the highest clinical net benefit.

**Conclusion:**

The FD model showed favorable diagnostic performance for AD. Among MLMs, the MoCA + FD model can predict AD with the highest efficiency and could be used as a non-invasive diagnostic method.

## 1 Introduction

Alzheimer’s disease (AD) is a common degenerative neurological disorder caused by the loss of function and death of neurons. Dementia is expected to affect 153 million people by 2050 (Nichols et al., 2022). After diagnosis, the average lifespan is just 4–8 years (Cui et al., 2019). Therefore, it is imperative to have an accurate diagnosis of AD.

Neuroimaging is essential for AD assessment, such as diffusion magnetic resonance imaging (dMRI), functional magnetic resonance imaging (fMRI), positron emission tomography (PET), and structural magnetic resonance imaging (SMRI) (Balaji et al., 2023). The PET scan is too expensive to be popularized. There are no uniform standards for the acquisition and post-processing of fMRI and dMRI. SMRI has received more research focus with better stability and repeatability compared to fMRI/dMRI (Cao et al., 2022). Three-dimensional (3D) T1-weighted has become a popular method to detect subtle changes in the brain (Ya et al., 2022).

In addition to conventional structure volume, patients with AD also exhibit brain cortical atrophy in the frontal and temporal cortices (Morys et al., 2002). Cortical atrophy is even found in the preclinical stages of AD, and involvement of the lateral aspects of the temporal pole, posterior cingulate gyrus, and frontal lobe might indicate a more rapid disease progression. Baron et al. (2001) suggested that patients with early-stage AD exhibit symmetric atrophy in both the left and right hemispheres. Moreover, the decline in memory function in AD is associated with specific regions of cortical atrophy. For example, AD-related deficits in recent memory and delayed recall are associated with atrophy in the entorhinal cortex (Di Paola et al., 2007). However, volumetric assessment cannot capture the inherent structural complexity of cortical atrophy. This complexity can be studied using cortical complexity, which describes the degree of complexity of objects that exhibit self-similarity within an appropriate spatial scale range (Pantoni et al., 2019).

**Figure 1:**
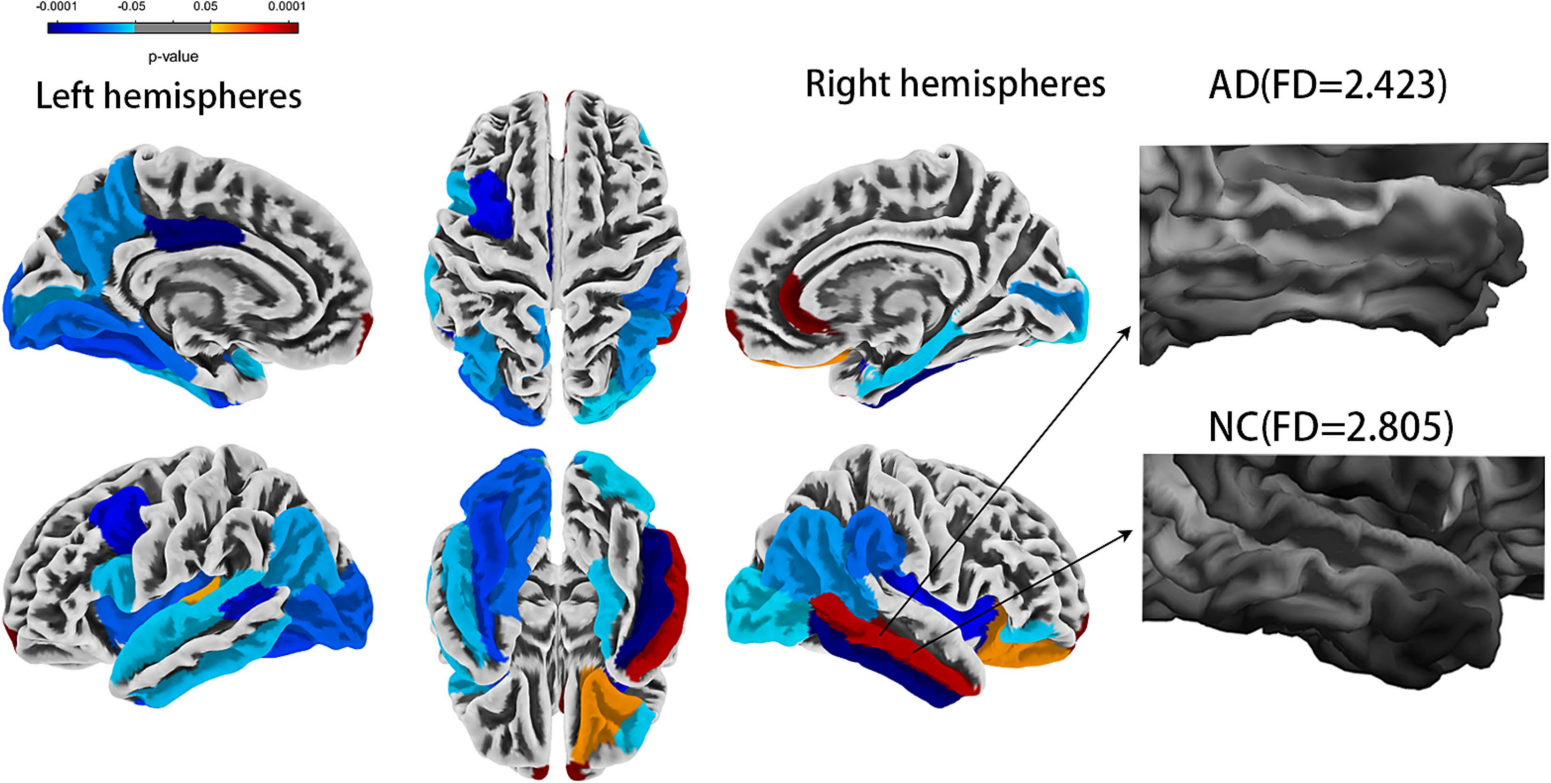
Comparison of whole brain fractal dimension between AD and NC groups [false discovery rate (FDR) corrected]. AD, Alzheimer’s disease; NC, normal cognitive function controls.

Cortical complexity reflects a cortical folding pattern. The cortices of AD patients appear smoother, indicating lower cortical complexity, while the cortices of normal cognitive (NC) function are more irregular indicating higher cortical complexity (Figure 1). Cortical complexity can be measured using fractal dimension (FD). FD is used to describe the shape complexity of irregular. As an index of cortical complexity, FD is a compact, unitless geometric shape characteristic that represents the amount of space an object occupies and yields a single quantitative measure of the object’s structural complexity (Stamatakis et al., 2016). The FD of brain gray matter (GM) can be calculated using commonly available high-resolution T1-weighted images, thus eliminating the need for additional magnetic resonance imaging (MRI) acquisitions. FD might help to quantify changes in the brain structure in patients with AD and could potentially help to identify patterns of brain atrophy in patients with AD (King et al., 2010). Compared to volume assessments and certain cortical morphological features, FD might have greater accuracy and sensitivity in the elderly, which might represent a new method to explore the neuropathological mechanism of AD (Pantoni et al., 2019; Nicastro et al., 2020; D'Antonio et al., 2022), with smaller variances and fewer sex effects (Wu et al., 2010). Despite progress in the rapid and rigorous diagnosis of AD, personalized diagnosis of AD remains a significant challenge (Qin et al., 2022).

To improve the predictability and feature interpretability for AD, machine learning models (MLMs) have been applied in AD prediction. Thus far, many biomarkers or genetic markers-based machine learning (ML) have been reported with varying results in response to different ML methods. Chang developed a Convolutional Neural Network (CNN) model with amyloidβ (Aβ), the prediction accuracy of mild cognitive impairment (MCI) was 84.2%, while another study using a support vector machine (SVM) model only reached 68% accuracy (Chang et al., 2021). Cullen N considered that Aβ biomarkers or apolipoprotein E (APOE) ε4 genotype did not contribute to the prediction of AD conversion (Cullen et al., 2022). It is still unclear whether biomarkers and genetic markers will affect the stability of MLMs. In addition, although FD-based ML has been widely applied for gliomas (Battalapalli et al., 2023), small vessel disease (Pantoni et al., 2019), Parkinson’s disease (Mo et al., 2022), and amyotrophic lateral sclerosis (Rajagopalan et al., 2023), scarce studies have indicated its application in the individualized diagnosis of patients with AD. Global cognitive function scales were applied to develop an MLM for the detection of AD (Goldstein et al., 2014; Wang B.-R. et al., 2019; Cai et al., 2023; Yi et al., 2023), but most of these studies lack external validation. There is a potential risk of overfitting without external validation.

Against this backdrop, we aim to develop various MLMs to find a more accurate and stable MLM for predicting AD by combining FD values, demographic characteristics, global cognitive function scales, biological markers, and genetic markers. Additionally, we further used Shapley additive explanation (SHAP) values, a united approach for MLMs, to rank the importance of input features, explain the results of the prediction model, and visualize individual variable predictions (Lee et al., 2024). The diagnostic performance of the optimal model was validated using an external validation cohort.

## 2 Materials and methods

### 2.1 Source data

The data used in this article were obtained from the Alzheimer’s Disease Neuroimaging Initiative (ADNI) database at the Laboratory of Neuro Imaging (LONI) Website.^1^ For the ADNI study, written informed consent was obtained from all participants. The institutional review board approved the study protocol at each participating center before protocol-specific procedures were performed. Taking 2021 as the cut-off time point, subjects were selected randomly from within a clinical database category (i.e., control and mild Alzheimer’s disease). All participants underwent MRI imaging acquired on 3 T scanners [Siemens (Munich, Germany)/GE (Boston, MA, USA)/Philips (Amsterdam, The Netherlands) Magnetom/Tim/Trio] using a magnetization-prepared rapid gradient echo (MPRAGE) T1-weighted sequence with the following parameters: thickness = 1.2 mm, time to echo (TE) 3.0–3.9 ms, repetition time (TR) 2,200–2,300 ms, flip angle = 9°, and isotropic voxels’ size = 0.9–1 mm^3^.

The exclusion criteria included loss of clinical data or the presence of image artifacts. For more detailed information, refer to: https://ida.loni.usc.edu/pages/access/studyData.jsp?categoryId=16.

For the study, 478 participants were selected, including 296 with NC function and 182 with AD. Demographic characteristics included age, sex, education, weight, heart rate, breath rate, temperature, and blood pressure. Montreal Cognitive Assessment (MoCA), Global Deterioration Scale (GDS), Functional Activities Questionnaire (FAQ), Neuropsychiatric Inventory (NPI), phospho-tau 181(p-tau 181), amyloidβ-42 (Aβ42)/amyloidβ-40 (Aβ40), apolipoprotein E (APOE) genotypes, and polygenic hazard score (PHS) were extracted for all participants. Additionally, 66 participants from our institution, including 33 with NC and 33 with AD served as an external validation cohort. The local Medical Research Ethics Committee approved this study. All participants gave their written informed consent before the study. These 66 participants underwent scans using the same parameters, along with the collection of identical biological markers and clinical and neuropsychological assessments.

### 2.2 Calculation of the FD

We conducted preprocessing of high-resolution T1-weighted images using the standard method in the Computational Anatomy Toolbox (CAT12)^2^ implemented in Statistical Parametric Mapping software (SPM12).^3^ The details of the procedures can be found in the CAT12 manual. Default settings were used throughout the analysis. The preprocessing steps included correction of bias-field inhomogeneities, segmentation into GM, white matter (WM), and cerebrospinal fluid, and normalization using the diffeomorphic anatomic registration through exponentiated lie algebra (DARTEL) algorithm. Following the CAT12 workflow described by Yotter et al. (2010), we estimated the FD of the cortex. Then, a spherical harmonic method was employed to reparametrize the cortical surface mesh based on an algorithm that reduces area distortions as a remedy for the topological defects. Finally, the approach of “spherical harmonic reconstructions” proposed by Yotter et al. (2010) was used to measure the local fractal dimensionality, which quantifies the cortical surface complexity. Mean FD values were calculated for 68 regions of interest (ROI), which were defined by the DK40 Atlas (Desikan et al., 2006), with standard procedures for ROI extraction as implemented in the CAT12 toolbox. The estimated FD values in each ROI were compared between the two groups. The statistical threshold was set at a false discovery rate (FDR) corrected value of *p* < 0.05. In summary, our analysis involved preprocessing of T1-weighted images, estimating cortical FD values using the CAT12 workflow, and reparametrization of the cortical surface using a spherical harmonic method.

### 2.3 Machine learning model development and validation

**Figure 2:**
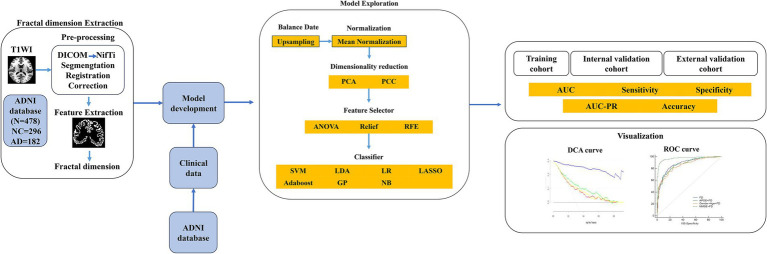
The flowchart of the machine learning steps. AD, Alzheimer’s disease; ADNI, Alzheimer’s Disease Neuroimaging Initiative; Aβ40, amyloidβ-40; Aβ42, amyloidβ-42; APOE, apolipoprotein E; ANOVA, analysis of variance; AUC, area under the ROC curve; DCA, decision curve analysis; DICOM, Digital Imaging and Communications in Medicine; NC, normal cognitive function; GP, Gaussian process; LASSO, least absolute shrinkage and selection operator; LDA, linear discriminant analysis; LR, logistic regression; NB, Naive Bayes; NifTi, Neuroimaging Informatics Technology Initiative; PCA, principal component analysis; PCC, Pearson correlation coefficient; PHS, polygenic hazard score; PR, precision-recall; RFE, recursive feature elimination; ROC, receiver operating characteristic; SVM, support vector machine.

The FD values incorporated into the MLM are abbreviated as FDs. FDs combined with demographic characteristics (including age, sex, education, weight, heart rate, breath rate, temperature, and blood pressure) as clinical data, along with MoCA, GDS, FAQ, NPI, p-tau 181, Aβ42/Aβ40, APOE*ε4, and PHS, were used to construct the combined models. The FD values with a significant difference (FDR-corrected *p* < 0.05) between AD and NC groups were selected to develop the FD model. Furthermore, FD and combined models were analyzed using FeAture Explorer (FAE v0.5.9)^4^ (Dimitriadis et al., 2020). To remove the imbalance of the training cohort’s dataset and to balance the positive/negative samples, we up-sampled by repeating random cases. We applied normalization to the feature matrix. Each feature vector was subtracted from the mean value of the vector and divided by its length. The dimension of the feature space was high; therefore, we applied the Pearson correlation coefficient (PCC) and principal component analysis (PCA) to the feature matrix. The feature vectors of the transformed feature matrix were independent of each other. Before building the model, we used three methods to select the features: recursive feature elimination (RFE), ANOVA, and Relief. They were commonly used methods to explore the significant features corresponding to the labels. The *F*-value was calculated to evaluate the relationship between features and the label. We sorted the features according to their corresponding *F*-values and selected a specific number of features to build the model. We used support vector machine (SVM), linear discriminant analysis (LDA), logistic regression (LR), least absolute shrinkage and selection operator (LASSO), AdaBoost, Gaussian process (GP), and Naive Bayes (NB) as the classifiers. To determine the model’s hyperparameters (e.g., the number of features), we applied 10-fold cross-validation on the training dataset. The hyperparameters were set according to the model performance on the validation dataset. The model’s performance was evaluated using receiver operating characteristic (ROC) curve analysis. The area under the receiver operating characteristic (ROC) curve (AUC) was calculated for quantification. The accuracy (Acc), sensitivity (Sen), specificity (Spe), and AUC precision-recall (AUC-PR) were also calculated. We also estimated the 95% confidence interval (CI) by bootstrapping with 1,000 samples. The best modeling approach was selected by comparing the other model’s highest AUC value and accuracy rate. One-stand error in FAE software was used to reduce the risk of overfitting. The flowchart of this study is shown in Figure 2.

### 2.4 Statistical analysis

Data were tested for normal distribution using the Kolmogorov–Smirnov test. Continuous variables were expressed as the mean ± standard deviation using a *t*-test. The non-normal distribution variables were expressed as median (interquartile range, IQR) and compared using a non-parametric test. The chi-squared and Fisher’s exact tests were used to compare categorical variables. All statistical analyses were two-sided, and a false discovery rate (FDR)-corrected *p* < 0.05 was considered statistically significant. All statistical analyses were performed using Statistical Package for the Social Sciences (SPSS) (version 26.0; Chicago, IL, USA). The CAT12 software in the SPM12 toolbox was used to compare FDs between the AD and NC groups. After FDR (*p* < 0.05) correction, the regions with statistical differences in FDs were obtained to develop machine learning models.

The performance of the model to predict AD was evaluated using the ROC. The ROC curve was plotted. The optimum threshold point of the ROC curve is determined using the Jorden index, and the Sen, Spe, Acc, AUC, and AUC-PR values were recorded to evaluate the diagnostic efficiency of each model. We used the DeLong test (DeLong et al., 1988) to compare the performances of the different models. Decision curve analysis (DCA) was used to compare the net benefits of various models at different threshold probabilities to increase the possibility of practical application in clinical practice. The decision curve was plotted using the “rmda” (risk model decision analysis) module of the R package (2020, R Core Team).^5^

### 2.5 Model explanation

We calculated the SHAP values to shed light on the model’s predictions. The SHAP method is an approach that could rank the importance of input features and explain the prediction model results (Hu et al., 2024). We used the SHAP summary bar plot and SHAP bees warm plot to visualize the contribution of each feature to the model’s predictions for specific instances. In contrast, the waterfall plot provides a detailed, step-by-step breakdown of how each feature moves the model’s output from the expected value to the actual prediction (Lee et al., 2024). All computations were executed using Python (version 3.12.2) and SHAP (version 0.42.1).

## 3 Results

### 3.1 Demographic and clinical characteristics

The demographic and clinical characteristics are shown in Table 1. In the ADNI cohort, only education, the whole-brain mean FD, MoCA, GDS, FAQ, p-tau 181, Aβ42/Aβ40, and PHS showed significant differences between the AD and NC groups (*p <* 0.05). There were much more significant differences between AD and NC groups in the external validation cohort except for age, weight, heart rate, breath rate, temperature, blood pressure, and APOE*ε4 (*p* > 0.05).

**Table 1:** Demographics and clinical characteristics of the participants.

Variables	ADNI cohort			External validation cohort		
	AD (*n* = 182)	NC (*n* = 296)	P	AD (*n* = 33)	NC (*n* = 33)	*p*-value
Age (years)	75.80 ± 8.20	75.86 ± 5.92	0.934	67.61 ± 10.36	62.36 ± 8.23	0.026^*^
Sex (M/F)	103/79	153/143	0.297	12/21	12/21	0.001^***^
Education (years)	16.00 (5.00)	16.00 (4.00)	0.002^**^	6.36 ± 4.93	9.18 ± 4.53	0.019^*^
Weight (kg)	71.81 ± 17.83	72.98 ± 19.37	0.508	76.48 ± 14.69	78.67 ± 16.85	0.575
Heart rate	64.59 ± 9.75	65.52 ± 11.79	0.356	65.33 ± 9.29	65.09 ± 10.09	0.919
Breath rate	16.00 (2.00)	16.00 (4.00)	0.467	16.30 ± 2.88	15.96 ± 2.42	0.613
Temperature (°C)	36.44 (0.59)	36.39 (0.57)	0.193	36.43 ± 0.36	36.4 ± 0.32	0.661
SBP (mm Hg)	132.35 ± 17.49	132.18 ± 15.88	0.916	132.67 ± 16.81	130.96 ± 16.01	0.676
DBP (mm Hg)	74.16 ± 9.06	73.53 ± 9.79	0.480	73.75 ± 9.93	72.48 ± 9.47	0.596
Whole-brain mean FD	2.56 ± 0.03	2.58 ± 0.03	0.001^***^	2.52 ± 0.02	2.54 ± 0.03	0.003^**^
MoCA	21.00 (6.00)	28.00 (3.00)	0.001^***^	8.33 ± 5.68	23.12 ± 5.50	0.001^***^
GDS	1.00 (1.25)	0.00 (1.00)	0.001^***^	1.00 (2.00)	0.00 (1.00)	0.007^**^
FAQ	11.47 ± 8.38	1.58 ± 4.42	0.001^***^	15.3 ± 5.39	0.15 ± 0.71	0.001^***^
NPI	1.00 (4.00)	0.00 (3.00)	0.219	6.00 (15.50)	0.00 (1.00)	0.001^***^
APOE*ε4 (Y/N)	128/54	83/213	0.191	11/22	9/24	0.592
p-tau 181	23.73 ± 8.16	16.51 ± 10.81	0.035^*^	/	/	/
Aβ42/Aβ40	0.17 ± 0.09	0.20 ± 0.08	0.001^***^	/	/	/
PHS	0.22 (0.23)	0.09 (0.07)	0.001^***^	/	/	/

Aβ40, amyloidβ-40; Aβ42, amyloidβ-42; APOE, apolipoprotein E; ADNI, Alzheimer’s Disease Neuroimaging Initiative; DBP, diastolic blood pressure; FAQ, Functional Activities Questionnaire; FD, fractal dimension; GDS, Global Deterioration Scale; MoCA, Montreal Cognitive Assessment; NC, normal cognitive; NPI, Neuropsychiatric Inventory; polygenic hazard score; PHS; p-tau 181, phospho-tau 181; SBP, systolic blood pressure. Continuous variables were expressed as the mean ± standard deviation. The non-normal distribution variables were expressed as median (interquartile range, IQR). *p < 0.05; **p < 0.01; ***p < 0.001.

### 3.2 Brain cortical complexity alterations in AD

The statistical analysis of the FDs from 69 regions revealed 30 regions that showed a significant difference (all FDR-corrected *p <* 0.05), including in the left hemisphere (banks superior temporal sulcus, inferior parietal cortex, inferior temporal gyrus, lateral occipital cortex, insula, frontal pole, para hippocampal, peri calcarine cortex, superior temporal gyrus, caudal middle frontal gyrus, fusiform gyrus, pars opercularis, posterior-cingulate cortex, lingual gyrus, transverse temporal, precuneus gyrus) and right hemisphere (banks superior temporal sulcus, inferior parietal cortex, inferior temporal gyrus, lateral occipital cortex, insula, frontal pole, para hippocampal, pericalcarine cortex, middle temporal gyrus, rostral anterior cingulate cortex, supramarginal gyrus, pars orbitalis, entorhinal cortex, and lateral orbital frontal cortex) (Figure 1 and Table 2). A univariate ROC curve was chosen to analyze the AUC of FDs from each brain region (Table 2).

**Table 2:** Fractal dimension values with differences between the AD and NC groups.

Left hemispheres	AUC (95% CI)	P (FDR)	Right hemispheres	AUC (95% CI)	P (FDR)
L-banks superior temporal sulcus	0.610 (0.558–0.662)	0.001	R-banks superior temporal sulcus	0.586 (0.534–0.639)	0.015
L-inferior parietal cortex	0.585 (0.533–0.637)	0.015	R-inferior parietal cortex	0.605 (0.552–0.659)	0.015
L-inferior temporal gyrus	0.582 (0.529–0.635)	0.032	R-inferior temporal gyrus	0.643 (0.591–0.695)	0.001
L-lateral occipital cortex	0.557 (0.504–0.611)	0.004	R-lateral occipital cortex	0.565 (0.512–0.619)	0.024
L-insula	0.629 (0.576–0.682)	0.004	R-insula	0.633 (0.582–0.685)	0.001
L-frontal pole	0.683 (0.634–0.732)	0.001	R-frontal pole	0.631 (0.581–0.681)	0.001
L-para hippocampal	0.537 (0.483–0.591)	0.005	R-para hippocampal	0.536 (0.483–0.589)	0.039
L-pericalcarine cortex	0.560 (0.507–0.614)	0.028	R-pericalcarine cortex	0.535 (0.413–0.518)	0.012
L-superior temporal gyrus	0.568 (0.514–0.621)	0.038	R-middle temporal gyrus	0.600 (0.547–0.653)	0.001
L-caudal middle frontal gyrus	0.617 (0.565–0.669)	0.001	R-rostral anterior cingulate cortex	0.639 (0.587–0.691)	0.001
L-fusiform gyrus	0.606 (0.555–0.658)	0.002	R-supramarginal gyrus	0.626 (0.575–0.677)	0.005
L-pars opercularis	0.610 (0.558–0.662)	0.030	R-pars orbitalis	0.540 (0.487–0.593)	0.042
L-posterior cingulate cortex	0.726 (0.680–0.772)	0.001	R-entorhinal cortex	0.589 (0.536–0.642)	0.038
L-lingual gyrus	0.605 (0.551–0.658)	0.005	R-lateral orbitofrontal cortex	0.553 (0.498–0.607)	0.009
L-transverse temporal	0.569 (0.517–0.621)	0.017			
L-precuneus gyrus	0.568 (0.514–0.622)	0.015			

AD, Alzheimer’s Disease; AUC, area under the receiver operating characteristic (ROC) curve; NC, normal cognitive; FDR, false discovery rate.

### 3.3 Model establishment

The machine learning models included the FD model and other combined models. The combined models were established using FD and clinical data, cognitive function scales, biological indicators, and genetic indicators. The detailed constituent factors and pipelines of all models are shown in Table 3. The feature distribution is shown in Supplementary Figure S1.

**Table 3:** Selected features for model construction.

Feature origin (*N*)^a^	Model^b^ (normalization/dimension reduction/feature selector/classifier)	Feature names
FD features	Mean/PCC/Relief/SVM	L-posterior cingulate cortex
(*N* = 10)		R-rostral anterior cingulate cortex
		L-frontal pole
		R-supramarginal gyrus
		R-inferior temporal gyrus
		L-parahippocampal
		L-pars opercularis
		R-middle temporal gyrus
		L-pericalcarine cortex
		R-pericalcarine cortex
Clinic + FD	Mean/PCA/RFE/NB	PCA_feature_2
(*N* = 9)		PCA_feature_3
		PCA_feature_5
		PCA_feature_7
		PCA_feature_9
		PCA_feature_13
		PCA_feature_29
		PCA_feature_31
		PCA_feature_32
MoCA+FD	Mean/PCC/ANOVA/GP	MoCA
(*N* = 5)		L-frontal pole
		L-posterior cingulate cortex
		R-insula
		R-inferior temporal gyrus
GDS + FD	Mean/PCC/Relief/SVM	L-posterior cingulate cortex
(*N* = 6)		L-frontal pole
		R-rostral anterior cingulate cortex
		GDS
		L-pars opercularis
		R-pericalcarine cortex
FAQ + FD	Mean/PCA/Relief/LR	PCA_feature_1
(*N* = 4)		PCA_feature_8
		PCA_feature_9
		PCA_feature_7
NPI + FD	Mean/PCA/ANOVA/GP	PCA_feature_1
(*N* = 6)		PCA_feature_8
		PCA_feature_19
		PCA_feature_10
		PCA_feature_29
		PCA_feature_15
APOE + FD	Mean/PCA/ANOVA/LR	PCA_feature_2
(*N* = 5)		PCA_feature_22
		PCA_feature_9
		PCA_feature_19
		PCA_feature_11
p-tau + FD	Mean/PCC/RFE/GP	p-tau
(*N* = 10)		L-frontal pole
		L-pericalcarine cortex
		L-pars opercularis
		L-posterior cingulate cortex
		R-supramarginal gyrus
		R-entorhinal cortex
		R-frontal pole
		R-insula
		R-rostral anterior cingulate cortex
Aβ42/Aβ40 + FD	Mean/PCC/RFE/SVM	Aβ42/Aβ40
(*N* = 7)		L-frontal pole
		L-pars opercularis
		L-posterior cingulate cortex
		R-inferior temporal gyrus
		R-insula
		R-rostral anterior cingulate cortex
PHS + FD	Mean/PCA/ANOVA/GP	PCA_feature_1
(*N* = 4)		PCA_feature_3
		PCA_feature_7
		PCA_feature_10

Aβ40, amyloidβ-40; Aβ42, amyloidβ-42; ANOVA, analysis of variance; APOE, apolipoprotein E; FD, fractal dimension; GP, Gaussian process; LASSO, least absolute shrinkage and selection operator; LDA, linear discriminant analysis; LR, logistic regression; NB, Naive Bayes; p-tau, phospho-tau; PCA, Principal component analysis; PCC, Pearson correlation coefficient; PHS, polygenic hazard score; RFE, recursive feature elimination; SVM, support vector machine. ^a^The total number of features in a distinct group. ^b^The processing of valid data features for modeling was called a model. ^c^ClinicFD features included FDs, age, sex, education, weight, heart rate, breath rate, temperature, and blood pressure.

### 3.4 Model evaluation results

**Figure 3:**
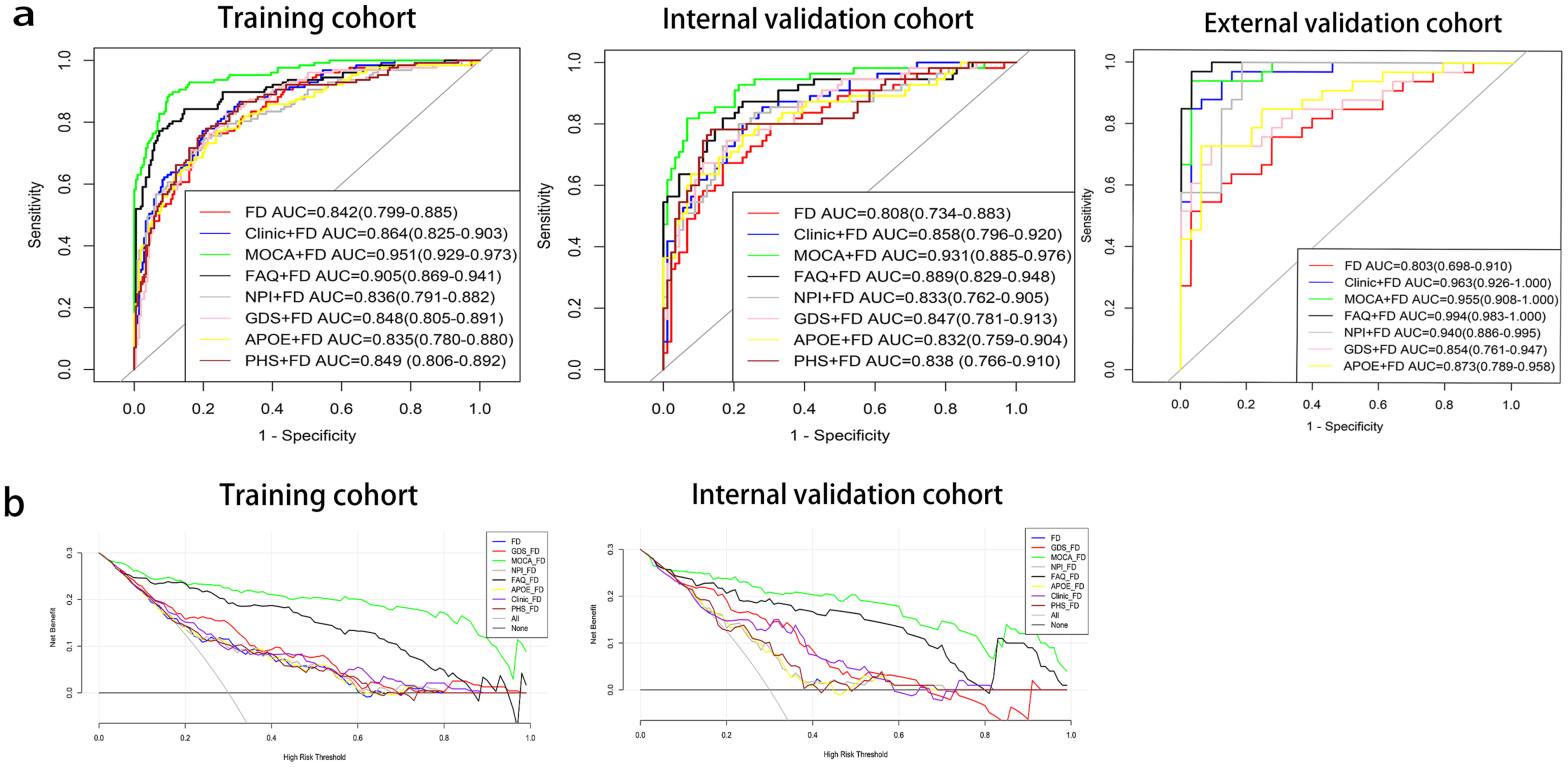
Receiver operating characteristic (ROC) curves **(a)** and decision curve analysis (DCA) curves **(b)** of prediction models. Aβ40, amyloidβ-40; Aβ42, amyloidβ-42; APOE, apolipoprotein E; FAQ, Functional Activities Questionnaire; FD, fractal dimension; GDS, Geriatric Depression Scale; MoCA, Montreal Cognitive Assessment; NPI, Neuropsychiatric Inventory; PHS, polygenic hazard score.

The MoCA + FD model shows the most vital ability to discriminate AD and NC in the training cohort (0.951 [95% CI: 0.929–0.973]) and internal validation cohort (0.931 [95% CI: 0.885–0.976]) (Table 4 and Figure 3A). It also showed superior performance in predicting AD in the external validation cohort. Among 34 participants with NC predicted using the MoCA + FD model, 32 (94.1%) were confirmed. In addition, among 32 participants with AD predicted by the MoCA + FD model, 31 participants (96.8%) were confirmed (Supplementary Figure S2). Overall, the MoCA + FD model achieved a favorable AUC of 0.955 ([95% CI: 0.908–1.0]) in the prospective validation cohort. In addition, the MoCA + FD model also had the highest precision-recall AUC of 0.979. The AUCs of the combined models were slightly higher than the standalone FD model across all cohorts. The MOCA + FD model showed the highest diagnostic performance for the cognitive function scale combined models in all cohorts. The results demonstrated that all models were reliable, with no overfitting. The p-tau + FD, Aβ42/Aβ40 + FD, and PHS + FD models also demonstrated slightly higher performance than the standalone FD model in both the training and internal validation cohorts. However, we did not conduct an external validation cohort due to the inconsistency of detection methods and different orders of magnitude.

**Table 4:** Detailed performance of the predictive models in all cohorts.

	Training cohort	Internal validation cohort	External validation cohort
FD			
AUC*	0.842 (0.799–0.885)	0.808 (0.734–0.883)	0.803 (0.698–0.910)
Sensitivity	0.740	0.709	0.515
Specificity	0.802	0.753	0.970
AUC-PR	0.784	0.728	0.834
Acc	0.778	0.736	0.742
Clinic + FD			
AUC*	0.864 (0.825–0.903)	0.858 (0.796–0.920)	0.963 (0.926–1.000)
Sensitivity	0.764	0.800	0.848
Specificity	0.802	0.820	0.969
AUC-PR	0.807	0.802	0.964
Acc	0.787	0.778	0.909
MoCA + FD			
AUC*	0.951 (0.929–0.973)	0.931 (0.885–0.976)	0.955 (0.908–1.0)
Sensitivity	0.882	0.818	0.939
Specificity	0.903	0.910	0.970
AUC-PR	0.936	0.917	0.979
Acc	0.895	0.875	0.955
GDS + FD			
AUC*	0.848 (0.805–0.891)	0.847 (0.781–0.913)	0.854 (0.761–0.947)
Sensitivity	0.709	0.709	0.727
Specificity	0.822	0.832	0.909
AUC-PR	0.757	0.799	0.888
Acc	0.778	0.785	0.818
FAQ + FD			
AUC*	0.905 (0.869–0.941)	0.889 (0.829–0.948)	0.994 (0.983–1.000)
Sensitivity	0.764	0.636	0.909
Specificity	0.927	0.933	0.970
AUC-PR	0.882	0.875	0.994
Acc	0.865	0.819	0.939
NPI + FD			
AUC*	0.836 (0.791–0.882)	0.833 (0.762–0.905)	0.940 (0.886–0.995)
Sensitivity	0.740	0.746	1.000
Specificity	0.802	0.798	0.818
AUC-PR	0.795	0.794	0.936
Acc	0.778	0.778	0.909
APOE + FD			
AUC*	0.835 (0.780–0.880)	0.832 (0.759–0.904)	0.873 (0.789–0.958)
Sensitivity	0.759	0.764	0.727
Specificity	0.772	0.742	0.939
AUC-PR	0.791	0.809	0.890
Acc	0.764	0.750	0.833
p-tau + FD			
AUC*	0.851 (0.795–0.906)	0.847 (0.770–0.925)	
Sensitivity	0.662	0.500	
Specificity	0.901	0.885	
AUC-PR	0.737	0.717	
Acc	0.833	0.778	
Aβ42/Aβ40 + FD			
AUC*	0.849 (0.805–0.894)	0.841 (0.772–0.911)	
Sensitivity	0.610	0.660	
Specificity	0.905	0.889	
AUC-PR	0.800	0.797	
Acc	0.792	0.802	
PHS + FD			
AUC*	0.849 (0.806–0.892)	0.838 (0.766–0.910)	
Sensitivity	0.740	0.782	
Specificity	0.816	0.865	
AUC-PR	0.789	0.809	
Acc	0.787	0.833	

Aβ40, amyloidβ-40; Aβ42, amyloidβ-42; APOE, apolipoprotein E; AUC, area under the receiver operating characteristic (ROC) curve; AUC-PR, AUC precision-recall; Acc, accuracy; FAQ, Functional Activities Questionnaire; FD, fractal dimension; GDS, Global Deterioration Scale; MoCA, Montreal Cognitive Assessment; NPI, Neuropsychiatric Inventory; p-tau, phospho-tau; PHS, polygenic hazard score. ^*^Data are the mean (95% CI).

The DeLong test was used to compare the diagnosis efficiency of the various models. When applying Delong’s test to compare the AUC values of each model, it was found that there were no statistically significant differences in the AUC values between the FD model and the other combined models in both the training and internal validation cohorts except for the MoCA + FD and FAQ + FD models (*p* > 0.05). The MoCA + FD model was superior to all other models in both the training and internal validation cohorts (*p* < 0.05). In the external validation cohort, the AUC values of all the models were above 0.8 (Supplementary Tables S1–S3). The FD model performed slightly worse, possibly due to the small sample size.

The DCA curves of the seven diagnostic models showed that within a larger threshold probability range, the MoCA + FD combined models had the highest clinical net benefit in both the training and internal validation cohorts (Figure 3B).

### 3.5 SHAP value

**Figure 4:**
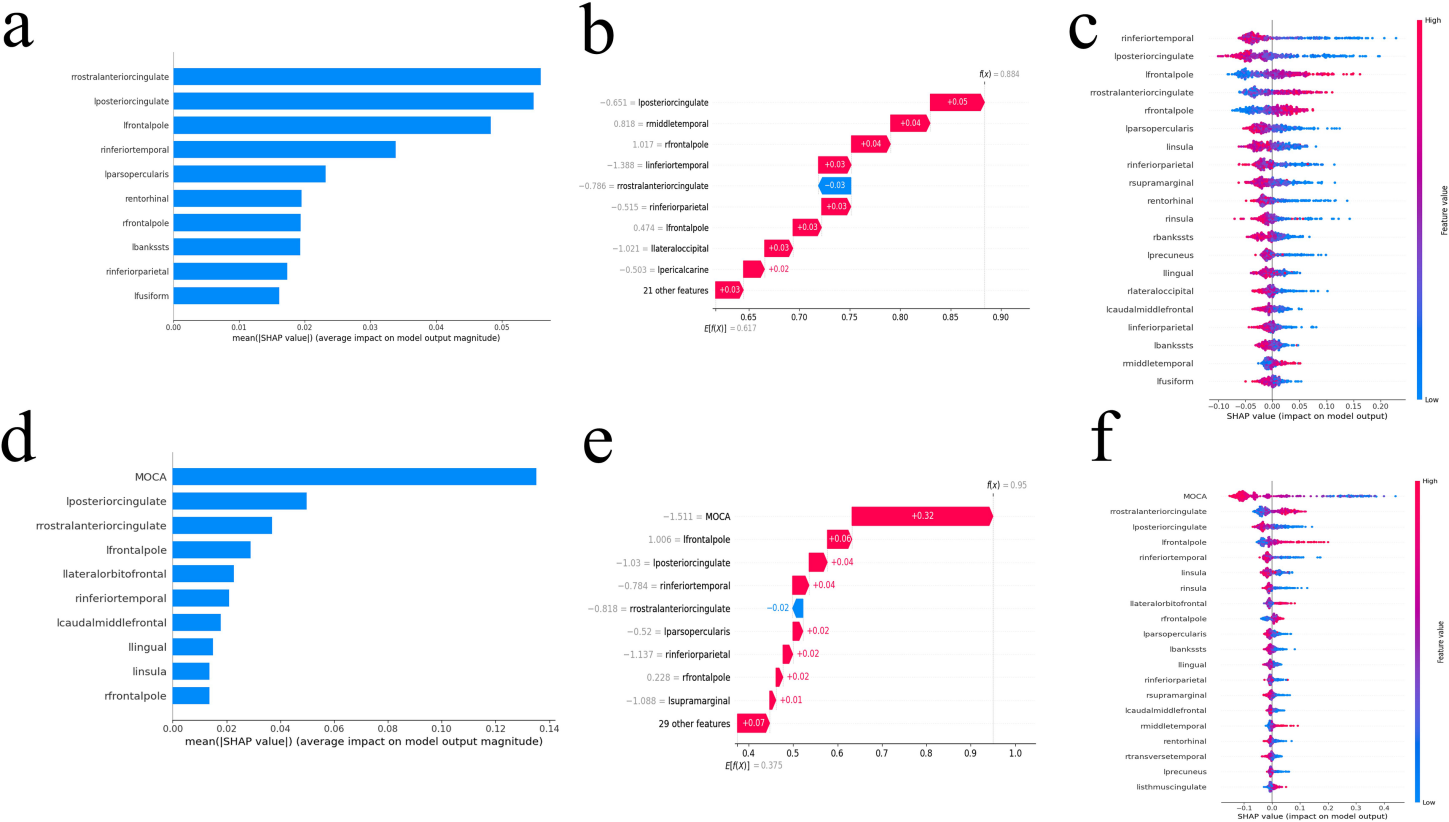
The SHAP value of the FD model **(a–c)** and MoCA + FD model **(d–f)**. SHAP summary bar plot **(a,d)**, SHAP bees warm plot **(c,f)**, and SHAP waterfall plot **(b,e)**. FD, fractal dimension; MoCA, Montreal Cognitive Assessment; SHAP, Shapley additive explanation.

Global explanation described the overall functionality of the model. As shown in SHAP summary plots and bees warm plot, the contributions of the feature to the model were evaluated using the average SHAP values and exhibited in descending order. In the FD model, the right rostral anterior cingulate cortex, left posterior cingulate cortex, and left frontal pole stood out (Figures 4A,C). In the MoCA + FD model, the MoCA, left posterior cingulate cortex, and right rostral anterior cingulate cortex stood out (Figures 4D,F). *E*[*f*(*x*)] refers to the average predicted output of the model across the entire dataset, providing insights into the model’s overall prediction tendency. In the FD model, among the variables, the left posterior cingulate cortex boosted the prediction by 0.05 and was ranked as the most influential factor (Figure 4B). In the MoCA + FD model, the MoCA was the most influential factor (Figure 4E). Additionally, the SHAP dependence plot elucidates how a single feature affects the output of the prediction model. The real values versus the SHAP values of these 10 features are shown in Supplementary Figure S3, and SHAP values higher than zero correspond to a positive class prediction in the model, in other words, a higher risk of AD.

## 4 Discussion

This study has observed significant alteration of brain cortical complexity in AD. Furthermore, as a new indicator, FD exhibits good and stable diagnostic performance when constructing MLMs for AD prediction. The diagnostic performance was further improved using the combined model. The MoCA + FD model exhibited the best diagnostic efficacy and highest net benefits compared to other combined models. The reliability and absence of overfitting of these optimal models were verified using an external validation cohort.

FD is one of the characteristic parameters used to describe structural complexity. Nicastro et al. (2020) demonstrated that the FD of the cortical complexity is a promising imaging tool to assess specific morphological patterns of GM damage in degenerative conditions, and the FD in disease-related regions was also associated with the severity of cognitive impairment (Nicastro et al., 2020). We found that FDs from 69 regions revealed that 30 regions showed a significant difference. Some of these regions were affected in both hemispheres, including the banks of the superior temporal sulcus, inferior parietal cortex, inferior temporal gyrus, lateral occipital cortex, frontal pole, insula, parahippocampal and pericalcarine cortex. These regions were primarily associated with memory, visual processing, olfaction, response to somatosensory stimuli, and emotional cognition, which were consistent with the common symptoms of AD. This indicated that these 8 pairs of FD indicators exhibit relatively significant changes in AD progression and should be closely monitored in clinical research, especially when comparing changes in values between the left and right hemispheres. These findings are consistent with those of previous studies (D'Antonio et al., 2022; Hason and Krishnan, 2022). In addition, the SHAP values indicated that the left posterior cingulate cortex and right rostral anterior cingulate cortex could be the key regions of AD. Additionally, we found that the left hemisphere had more regions than the right hemisphere, which is consistent with previous studies (Sandu et al., 2014; Jao et al., 2021). This could be attributed to cortical surface shape with a rightward complexity asymmetry (King et al., 2010). Unlike previous studies (Qin et al., 2022; Ya et al., 2022), removing redundant features of the entire brain could enhance the classification performance of the model (Liu et al., 2015). The regions with statistical differences in FDs were obtained to develop the FD model. This approach helped to improve the predictive performance of the FD model and avoid overfitting.

A previous study Chiu et al. (2022) combined 3 demographic features, 1 clinical feature, 18 brain-image features, and 3 plasma biomarkers to develop a machine learning model for predicting AD, NC, and MCI. Although the AUC was higher than 0.85, many enrolled features reduced the interpretability of the model. In a parallel study, several scholars carried out similar work (Wang Y. et al., 2019; Khatri and Kwon, 2022) by combining all features into a single model, which resulted in unclear clinical applicability. Usually, not all participants can complete all the tests, which is time-consuming and impractical. We tend to consider that the simpler the machine learning model, the more feasible and interpretable it is. Compared to previous studies, to increase interpretable clinical applicability, we combined demographic characteristics, global cognitive function scales, and biological markers with FDs separately. This study found that the diagnostic performance improved with the MoCA + FD and FAQ + FD models, which also exhibited excellent predictive performance in the external validation cohort. Several factors contribute to this improvement. First, both MoCA and FAQ showed statistically significant differences between the NC and AD groups, which helped to improve the diagnostic performance. Second, these indicators correlate with the occurrence and AD progression. Previous studies have shown that MoCA and FAQ are sensitive indicators for diagnosing AD (Goldstein et al., 2014; Wang B.-R. et al., 2019). Yi et al. (2023) found that FAQ was associated with a higher risk of AD onset, with the AUC of MLMs reaching 0.91 when using XGBoost as the classifier. Cai et al. (2023) combined MoCA, clinical, and MRI features to construct MLMs, with the AUC of this model reaching 0.853 in predicting early AD.

Additionally, we chose APOE*ε4, PHS, p-tau181, and Aβ42/40 as machine learning features. Substantial evidence from clinical and basic research suggests that a major pathway through which APOE*ε4 and PHS increase the risk of AD has been identified (Raulin et al., 2022; Spencer et al., 2022; Vacher et al., 2022). Gao et al. (2022) found that the AUC of their machine learning model (which included p-tau, Aβ42/40, APOE, and MRI) ranged from 0.843 to 0.909, aligning with our findings. However, some previous studies showed different results. The AUCs in a previous study were all below 0.8 (Zhang et al., 2022), while another study reported an AUC of 0.96 (Park et al., 2023). We speculated that the diagnostic efficacy of machine-learning models based on biomarkers and genetic markers might not be stable (Leuzy et al., 2021). Although PHS, Aβ42/40, and p-taul81 were useful measures for monitoring neuropathy markers of cognitive decline, especially for AD (Moscoso et al., 2021), there is currently no uniform cut-off or unified detection method (Karikari et al., 2022). In addition, we found the AUCs of these MLMs did not significantly improve when compared to the FD model. Since the lack of unified detection methods limits the use of PHS, p-tau 181, and Aβ42/40, the results may be different, and we did not conduct external validation.

Notably, we observed that the AUC of the clinical + FD model was lower than other models in the training and internal validation cohorts, but the result of the external validation set was similar to other models. We speculated that inter-dataset clinical differences might exert an important impact. In the external validation cohort, AD participants from our institution presented severe cognitive impairment with lower MoCA scores than the ADNI cohort. These results also demonstrated the clinical features could not achieve the best performances. A previous study Apostolova et al. (2014) enrolled AD and NC subjects from the ADNI-1 cohort, and the AUC of the hippocampal volume + clinic model was the lowest among all combined models. Similar results were found in a recent study Chen et al. (2023).

As for the limitations, the sample size was relatively small for the external validation cohort, and patients in this cohort were mostly with severe cognitive impairment, which could influence part of the AUC in the external validation cohort. We will further increase the sample size in our center to address these problems. We will expand the sample size in future research to improve the analysis of the subtypes of patients with AD. Given the limitations in the completeness of clinical data from the ADNI database, we did not use more novel Alzheimer’s disease biomarkers and genetic markers. Finally, participants in the ADNI database were typically well-educated elderly individuals subject to a narrow scope of selection.

## 5 Conclusion

In conclusion, the brain regions with significant alteration of cortical complexity are expected to serve as potential neuroanatomical markers of AD. The MLMs based on FDs demonstrated sound diagnostic stability and efficiency for AD. FD combined with global cognitive function scales based on ML may prove an effective diagnosis method of AD with higher accuracy, as it reduces the unnecessary deployment of therapeutics and streamlines the workflow of clinicians.

## Data Availability

The datasets presented in this study can be found in online repositories. The names of the repository/repositories and accession number(s) can be found in the article/Supplementary material.

## Footnotes

^1^ https://adni.loni.usc.edu/

^2^ http://dbm.neuro.uni-jena.de/cat/

^3^ https://www.fil.ion.ucl.ac.uk/spm/

^4^ https://github.com/salan668/FAE

^5^ https://www.R-project.org/

## Supplementary material

Supplementary Figure S1 The feature distribution and ROC curves of multiple MLMs. FD, Fractal dimension; MOCA, Montreal Cognitive Assessment; FAQ, Functional Activities Questionnaire; NPI, Neuropsychiatric Inventory; GDS, Geriatric Depression Scale; Aβ40, Amyloidβ-40; Aβ42, Amyloidβ-42; APOE, apolipoprotein E; PHS, polygenic hazard score.

Supplementary Figure S2 The performance of multiple MLMs in external validation cohort. FD, Fractal dimension; MOCA, Montreal Cognitive Assessment; FAQ, Functional Activities Questionnaire; NPI, Neuropsychiatric Inventory; GDS, Geriatric Depression Scale; APOE, apolipoprotein E; PHS, polygenic hazard score.

Supplementary Figure S3 The SHAP dependence plot of FD model **(A)** and MOCA+FD model **(B)**. FD, Fractal dimension; MOCA, Montreal Cognitive Assessment; SHAP, Shapley Additive explanation
