# Supplementary material for: Machine learning models for diagnosing Alzheimer’s disease using brain cortical complexity
Source: Front Aging Neurosci. 2024 Oct 9;16:1434589. doi: 10.3389/fnagi.2024.1434589 (PMC11500324; doi:10.3389/fnagi.2024.1434589)
Supplement: Supplementary file 1 [file Table_1.docx]

Table S1 The p-values^a^ for pairwise comparisons of multiple MLMs in training cohort

|  | FD | Clinic+FD^a^ | MOCA+FD | GDS+FD | FAQ+FD | NPI+FD | APOE+FD | P-tau+FD | Aβ42/Aβ40+FD | PHS+FD |
| --- | --- | --- | --- | --- | --- | --- | --- | --- | --- | --- |
| FD |  | 0.154 | 0.001* | 0.729 | 0.001* | 0.701 | 0.629 | 0.099 | 0.809 | 0.905 |
| Clinic+FD^b^ | 0.154 |  | 0.001* | 0.458 | 0.016* | 0.071 | 0.082 | 0.589 | 0.932 | 0.530 |
| MOCA+FD | 0.001* | 0.001* |  | 0.001* | 0.004* | 0.001* | 0.001* | 0.002* | 0.004* | 0.021* |
| GDS+FD | 0.729 | 0.458 | 0.001* |  | 0.010* | 0.576 | 0.509 | 0.347 | 0.565 | 0.330 |
| FAQ+FD | 0.001* | 0.016* | 0.004* | 0.010* |  | 0.001* | 0.001* | 0.112 | 0.192 | 0.031* |
| NPI+FD | 0.701 | 0.071 | 0.001* | 0.576 | 0.001* |  | 0.877 | 0.953 | 0.693 | 0.217 |
| APOE+FD | 0.629 | 0.082 | 0.001* | 0.509 | 0.001* | 0.877 |  | 0.904 | 0.753 | 0.875 |
| P-tau+FD | 0.099 | 0.589 | 0.002* | 0.347 | 0.112 | 0.953 | 0.904 |  | 0.655 | 0.422 |
| Aβ42/Aβ40+FD | 0.809 | 0.932 | 0.004* | 0.565 | 0.192 | 0.693 | 0.753 | 0.655 |  | 0.822 |
| PHS+FD | 0.905 | 0.530 | 0.021* | 0.330 | 0.031* | 0.217 | 0.875 | 0.422 | 0.822 |  |

MLMs, machine learning models. FD, Fractal dimension; MOCA, Montreal Cognitive Assessment; FAQ, Functional Activities Questionnaire; NPI, Neuropsychiatric Inventory; GDS, Geriatric Depression Scale; Aβ40, Amyloidβ-40; Aβ42, Amyloidβ-42; APOE, apolipoprotein E.

^a^ The p-values of pairwise comparison by Delong’s test were reported.

^b^ Clinic+FD features included FDs, age, sex, education, weight, heart rate, breath rate, temperature, blood pressure.

^*^ Represents p < 0.05

Table S2 The p-values^a^ for pairwise comparisons of multiple MLMs in internal validation cohort

|  | FD | Clinic+FD | MOCA+FD | GDS+FD | FAQ+FD | NPI+FD | APOE+FD | P-tau+FD | Aβ42/Aβ40+FD | | PHS+FD |
| --- | --- | --- | --- | --- | --- | --- | --- | --- | --- | --- | --- |
| FD |  | 0.122 | 0.002* | 0.297 | 0.001* | 0.147 | 0.494 | 0.931 | 0.849 | 0.832 | |
| Clinic+FD^b^ | 0.122 |  | 0.010* | 0.122 | 0.326 | 0.623 | 0.294 | 0.212 | 0.541 | 0.499 | |
| MOCA+FD | 0.002* | 0.010* |  | 0.008* | 0.224 | 0.003* | 0.034* | 0.009* | 0.004* | 0.025* | |
| GDS+FD | 0.297 | 0.122 | 0.008* |  | 0.208 | 0.248 | 0.193 | 0.947 | 0.950 | 0.995 | |
| FAQ+FD | 0.001* | 0.326 | 0.224 | 0.208 |  | 0.009* | 0.007* | 0.006* | 0.003* | 0.313 | |
| NPI+FD | 0.147 | 0.623 | 0.003* | 0.248 | 0.009* |  | 0.834 | 0.324 | 0.252 | 0.402 | |
| APOE+FD | 0.494 | 0.294 | 0.034* | 0.193 | 0.007* | 0.834 |  | 0.356 | 0.908 | 0.916 | |
| P-tau+FD | 0.931 | 0.212 | 0.009* | 0.947 | 0.006* | 0.324 | 0.356 |  | 0.933 | 0.957 | |
| Aβ42/Aβ40+FD | 0.849 | 0.541 | 0.004* | 0.950 | 0.003* | 0.252 | 0.908 | 0.933 |  | 0.816 | |
| PHS+FD | 0.832 | 0.499 | 0.025* | 0.995 | 0.313 | 0.402 | 0.916 | 0.957 | 0.816 |  | |

MLMs, machine learning models. FD, Fractal dimension; MOCA, Montreal Cognitive Assessment; FAQ, Functional Activities Questionnaire; NPI, Neuropsychiatric Inventory; GDS, Geriatric Depression Scale; Aβ40, Amyloidβ-40; Aβ42, Amyloidβ-42; APOE, apolipoprotein E.

^a^ The p-values of pairwise comparison by Delong’s test were reported.

^b^ Clinic+FD features included FDs, age, sex, education, weight, heart rate, breath rate, temperature, blood pressure.

^*^ Represents p < 0.05

Table S3 The p-values^a^ for pairwise comparisons of multiple MLMs in external validation cohort

|  | FD | Clinic+FD | MOCA+FD | GDS+FD | FAQ+FD | NPI+FD | APOE+FD |
| --- | --- | --- | --- | --- | --- | --- | --- |
| FD |  | 0.005* | 0.001* | 0.218 | 0.001* | 0.022* | 0.151 |
| Clinic+FD^b^ | 0.005* |  | 0.602 | 0.032* | 0.103 | 0.430 | 0.027 |
| MOCA+FD | 0.001* | 0.602 |  | 0.010* | 0.255 | 0.240 | 0.016* |
| GDS+FD | 0.218 | 0.032* | 0.010* |  | 0.003* | 0.108 | 0.661 |
| FAQ+FD | 0.001* | 0.103 | 0.255 | 0.003* |  | 0.036* | 0.003* |
| NPI+FD | 0.022* | 0.430 | 0.240 | 0.108 | 0.036* |  | 0.109 |
| APOE+FD | 0.151 | 0.027 | 0.016* | 0.661 | 0.003* | 0.109 |  |

MLMs, machine learning models. FD, Fractal dimension; MOCA, Montreal Cognitive Assessment; FAQ, Functional Activities Questionnaire; NPI, Neuropsychiatric Inventory; GDS, Geriatric Depression Scale; Aβ40, Amyloidβ-40; Aβ42, Amyloidβ-42; APOE, apolipoprotein E.

^a^ The p-values of pairwise comparison by Delong’s test were reported.

^b^ Clinic+FD features included FDs, age, sex, education, weight, heart rate, breath rate, temperature, blood pressure.

^*^ Represents p < 0.05
