# Supplementary figures and images for: Machine learning models for diagnosing Alzheimer’s disease using brain cortical complexity
Source: Front Aging Neurosci. 2024 Oct 9;16:1434589. doi: 10.3389/fnagi.2024.1434589 (PMC11500324; doi:10.3389/fnagi.2024.1434589)

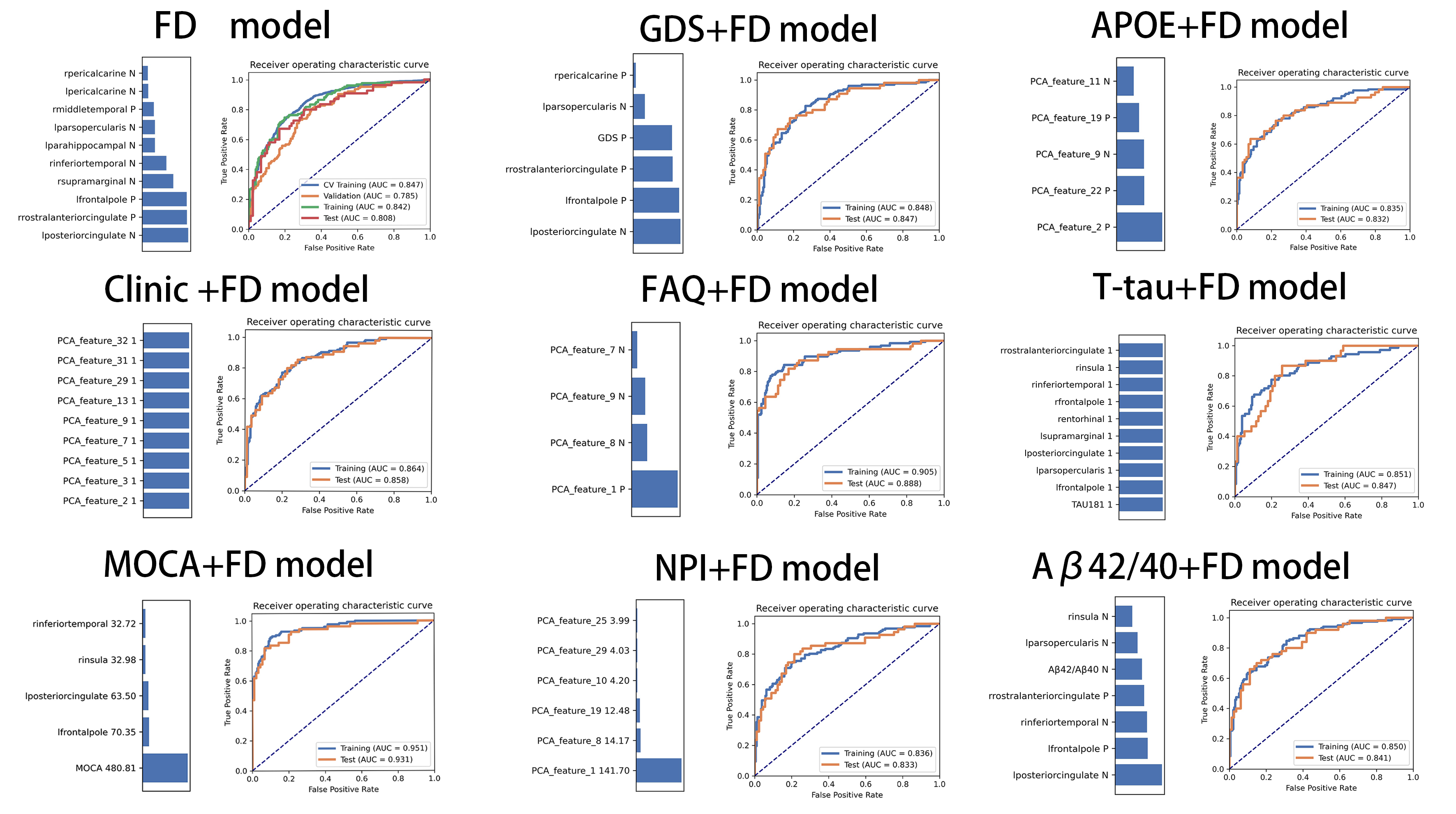

Supplement: Supplementary Figure S1 — The feature distribution and ROC curves of multiple MLMs. FD, Fractal dimension; MOCA, Montreal Cognitive Assessment; FAQ, Functional Activities Questionnaire; NPI, Neuropsychiatric Inventory; GDS, Geriatric Depression Scale; Aβ40, Amyloidβ-40; Aβ42, Amyloidβ-42; APOE, apolipoprotein E; PHS, polygenic hazard score. [file Image_1.TIF]

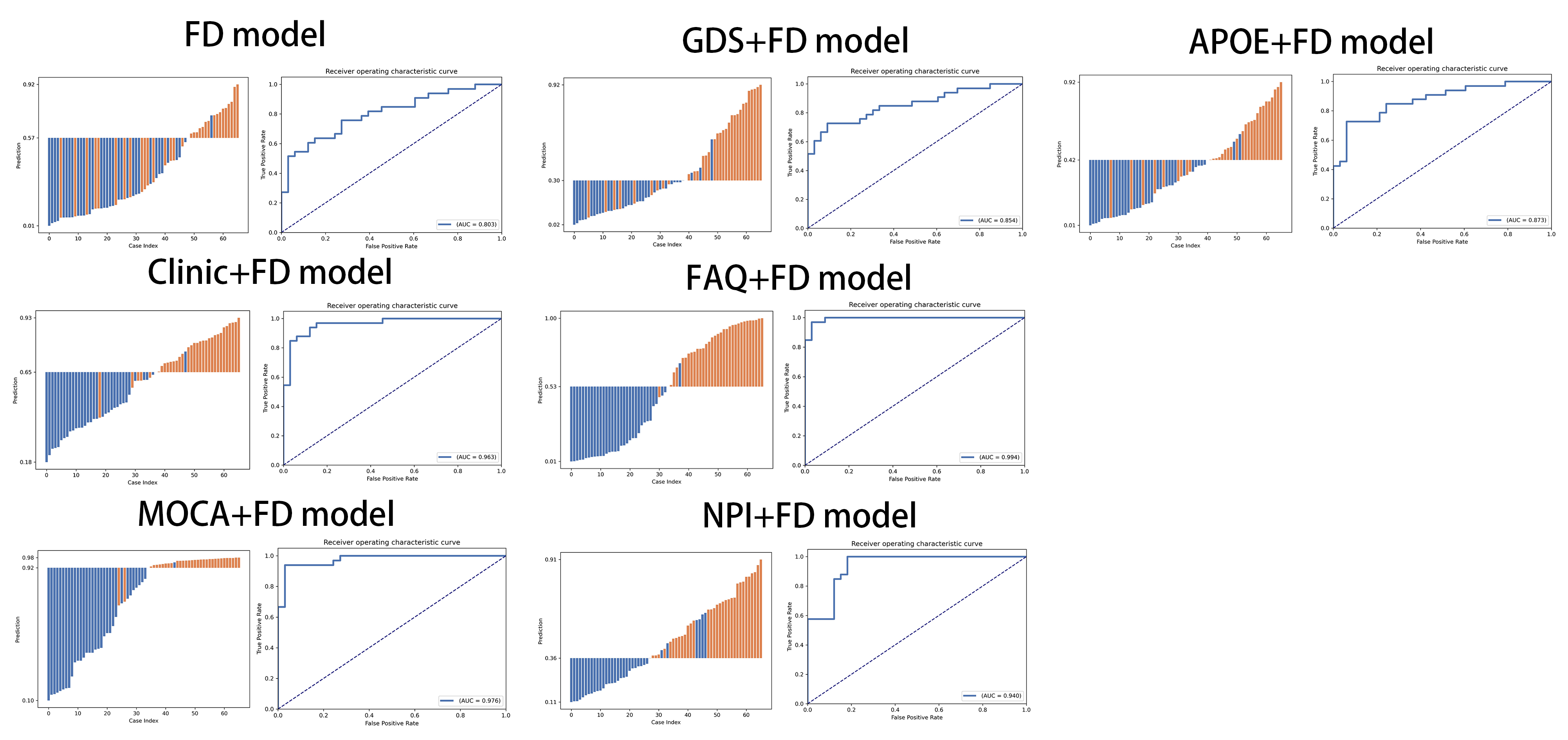

Supplement: Supplementary Figure S2 — The performance of multiple MLMs in external validation cohort. FD, Fractal dimension; MOCA, Montreal Cognitive Assessment; FAQ, Functional Activities Questionnaire; NPI, Neuropsychiatric Inventory; GDS, Geriatric Depression Scale; APOE, apolipoprotein E; PHS, polygenic hazard score. [file Image_2.TIF]

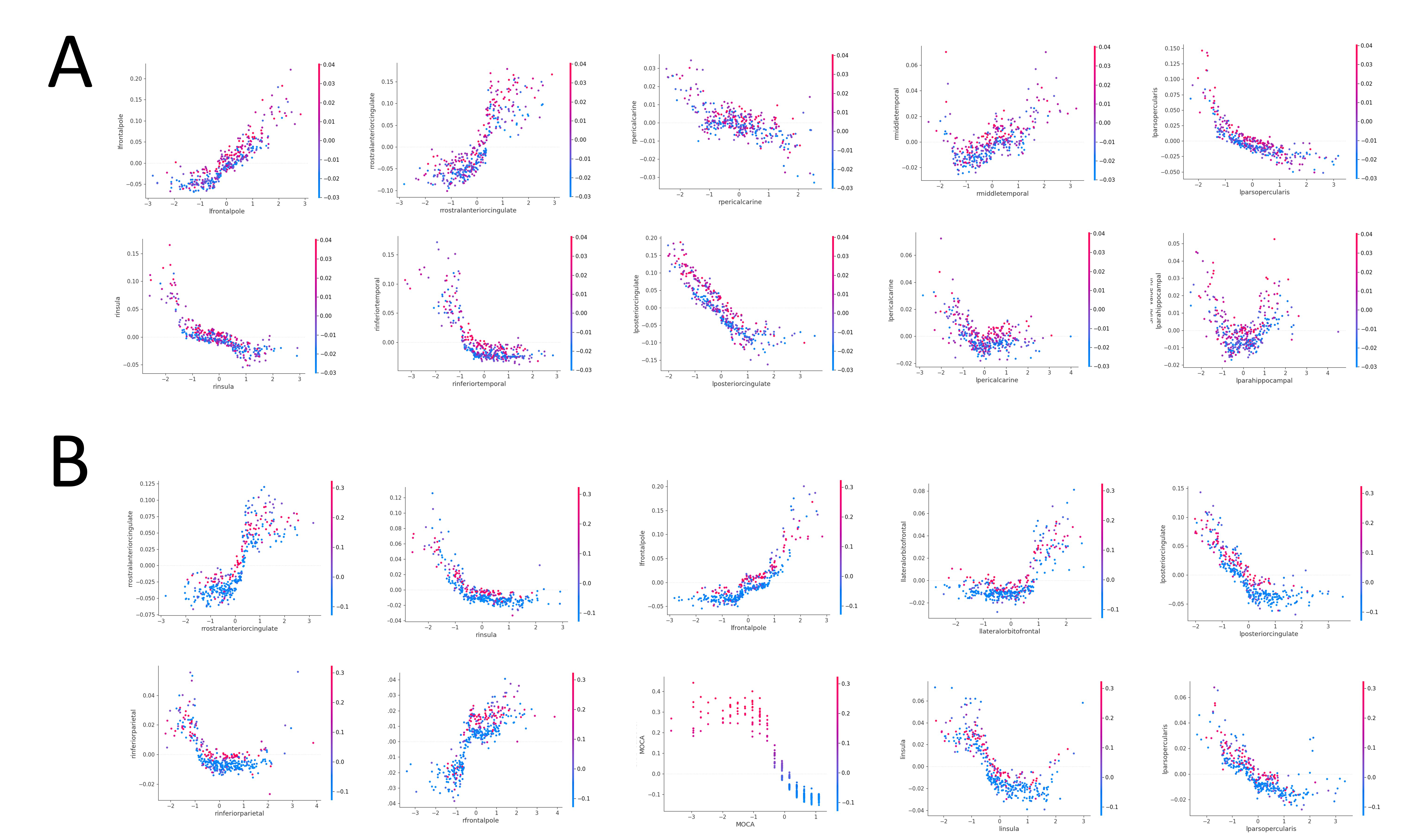

Supplement: Supplementary Figure S3 — The SHAP dependence plot of FD model (A) and MOCA+FD model (B). FD, Fractal dimension; MOCA, Montreal Cognitive Assessment; SHAP, Shapley Additive explanation [file Image_3.TIF]
